# Handgun purchasing characteristics and firearm suicide risk: a nested case–control study

**DOI:** 10.1186/s40621-021-00365-3

**Published:** 2021-12-13

**Authors:** Julia P. Schleimer, Rose M. C. Kagawa, Hannah S. Laqueur

**Affiliations:** 1 Violence Prevention Research Program, Department of Emergency Medicine, University of California, Davis, CA USA; 2 California Firearm Violence Research Center, 2315 Stockton Blvd., Sacramento, CA 95817 USA

**Keywords:** Suicide, Firearm, Handgun, Case–control

## Abstract

**Background:**

Firearms are the most lethal method of suicide and account for approximately half of all suicide deaths nationwide. We describe associations between firearm purchasing characteristics and firearm suicide.

**Methods:**

Data on all legal handgun transactions in California from 1996 to 2015 were obtained from the California Department of Justice Dealer’s Record of Sale database. Handgun purchasers were linked to mortality data to identify those who died between 1996 and 2015. To account for variation in timing and duration of observation time, analyses were stratified by birth cohort. The primary analysis focused on those aged 21–25 in 1996. A secondary analysis tested associations among those aged 50–54 in 1996. Using incidence density sampling, purchasers who died by firearm suicide (cases) were each gender-matched to 5 purchasers (controls) who remained at risk at the case’s time of death. We examined the characteristics of purchasers and transactions, focusing on the transaction closest in time to the case’s death. Data were analyzed with conditional logistic regression.

**Results:**

There were 390 firearm suicides among the younger cohort and 512 firearm suicides among the older cohort. Across both cohorts, older age at first purchase and the purchase of a revolver were associated with greater risk of firearm suicide. For example, among the younger cohort, those who purchased a revolver versus semiautomatic pistol had 1.78 times the risk of firearm suicide (95% CI 1.32, 2.40) in multivariable models. Other associations varied across cohorts, suggesting cohort or age effects in purchasing patterns.

**Conclusions:**

Findings add to the evidence on firearm suicide risk and may help inform prevention strategies and future research.

## Background

In 2019, 47,511 Americans died from suicide (https://www.cdc.gov/injury/wisqars/fatal.html), half of whom (23,941) used a firearm (https://www.cdc.gov/injury/wisqars/fatal.html). Firearms are the most lethal method of suicide; approximately 90% of attempts result in death (Conner et al. 2019). Researchers, practitioners, and policy makers have therefore been concerned with identifying individuals at increased risk for suicide and limiting their access to firearms, for example, through firearm prohibition, temporary firearm removal, or secure firearm storage (Swanson 2019; Pallin et al. 2019; Zeoli and Webster 2019). A central challenge is accurately identifying those at risk of firearm suicide and developing feasible and effective interventions. Indeed, firearm prohibitions based on mental illness—the category of prohibition arguably most applicable to suicide prevention—are not prohibiting to most people likely to die by firearm suicide (Swanson 2021).

Prior research has shown a clear association between the purchase of a firearm and risk for firearm suicide. For example, in a large cohort of Californians, firearm suicide risk in the month after handgun purchase was 100.1 (95% CI 55.8–179.9) times higher among handgun purchasers than it was among non-purchasers (Studdert et al. 2020). Risk among handgun purchasers remained high for the entire 12 years under study.

However, longitudinal data on firearm transaction records have not been systematically analyzed to determine whether specific characteristics of firearm purchasers and transactions can help identify important risk factors for firearm suicide. In this nested case–control study, we describe differences in handgun purchasing characteristics between purchasers who died by firearm suicide and those who did not. Our goal is to identify markers of risk that may help inform future research and new interventions to prevent firearm suicide.

## Methods

### Study population and cohort definitions

Our study population was drawn from a cohort of individuals who legally purchased a handgun in California between 1996 and 2015. We obtained data on handgun transactions from the California Department of Justice (CA DOJ) Dealer’s Record of Sale (DROS) database, which contains detailed information on all legal handgun transactions in the state.

Individuals entered the cohort at the time of their first purchase and were considered at risk until December 31, 2015, their death from any cause, or when they could no longer be identified as a resident of California, whichever came first. We identified deaths by linking purchasers to the California Department of Public Health’s Death Statistical Master File, matching on name, date of birth, and gender. California residence was determined by querying LexisNexis Public Records; purchasers were censored two and a half years after their last identifiable California address (the median length of time between moves among cohort members).

Because we had a dynamic cohort (i.e., people entered the cohort when they first purchased a handgun during the period 1996–2015 and exited the cohort when they died, left the state, or the study period ended), we did not observe everyone’s lifetime exposure (handgun purchasing) history. For example, for those older than age 21 (the legal age of handgun purchase in CA) (Code and §27505) in 1996, we had no information on whether they had purchased any handguns before 1996, and, if so, how many or what types. The amount of potential “missing” data increases with older purchaser age in 1996. The study period end date also truncates data on later life purchasing, and the degree of this truncation increases with younger purchaser age in 1996. These different types of “missing” data could introduce bias or impact generalizability, since purchasers had varying amounts of possible observation time, and age-specific observation time varied systematically with the risk of exposure and outcome, as firearm suicide risk increases with age (Wintemute 2015). A lack of data on pre-1996 purchases could also induce survivor bias if cases with certain exposures were less likely than other purchasers to survive to study enrollment (Hu et al. 2016). To minimize these biases, maximize what we might learn from the data, and avoid averaging over potentially heterogeneous associations, we created two sub-cohorts of purchasers: (1) those aged 21–25 in 1996 and (2) those aged 50–54 in 1996. For the younger cohort, we had complete purchasing data on people from (roughly) the age at which they were legally eligible to purchase a handgun, minimizing potential survivor bias in this cohort. As such, we consider the younger cohort our primary analysis and the older cohort a secondary analysis. The main benefit of the older cohort is that we observe them during higher risk ages for suicide. However, there is more missing data and greater potential for survivor bias in this cohort. Finally, it is worth noting that we did not match on age at time of entry into the study population; that is, an individual who was 21 in 1996 could have, for example, purchased their first handgun and entered the cohort at age 31. Results are reported separately for each cohort.

### Independent variables

We examined features of purchasers, transactions, and handguns. Purchaser characteristics included self-reported race and ethnicity (Asian, Black, Hispanic/Latinx, white, and other, which includes unknown or missing race/ethnicity), and age at first (observed) purchase. Transaction characteristics included whether the handgun was purchased at a gun show; whether the record was a sale versus other operation, including, for example, voluntary registration or collector’s report (these were rare in our sample); and the distance between the purchaser’s address and the location of the dealer. Handgun characteristics included category (revolver; semiautomatic pistol; or other, which included missing information); caliber, classified into small (e.g., .22, .25, .32), medium (e.g., .38, .380, 9 mm), and large (e.g., .40, .44, .45, which included a small number of other and unknown caliber) based on a classification schema developed in previous work (Wintemute et al. 1998; Wright et al. 2005); and whether the gun was inexpensive or not, proxied by the manufacturer, which we identified as producing handguns in the bottom quantile of prices listed in the *Blue Book of Gun Values* (Fushing and Roy 2018). Because we were most interested in proximal risk, if a purchaser had multiple purchases during the exposure period, we focused on the characteristics of the transaction and handgun pertaining to the purchase closest in time to the case’s death (the “index” purchase). We additionally created a continuous variable for the total number of purchases per person and a 3-level categorical variable for the amount of time between the index purchase and the purchase just prior, if any (the first category included purchasers who only had 1 purchase or whose index purchase was less than 2 years after their prior purchase; the second included purchasers whose index purchase was 2–4 years after their prior purchase; the third included purchasers whose index purchase was 5 or more years after their prior purchase).

We adjusted for purchasers’ community characteristics by geocoding their addresses recorded in the Dealer Record of Sale to identify their census tract of residence at each transaction. Community characteristics measured at the census-tract level included population density, percentage of the population under age 18, the ratio of males aged 15–34 years to females aged 15–34 years, percentages non-Hispanic/Latinx white and non-Hispanic/Latinx Black, and a socioeconomic index (i.e., the first principal component of the percentage of the population aged 25 and over with a bachelor’s degree or higher [reverse coded], the percentage of the population with past-year income below the poverty level, the percentage of households with public assistance income in the last year, the percentage of the population aged 16 + in the civilian labor force who were unemployed, and the percentage of households with children that were female-headed). All census-tract level covariates were obtained from Geolytics’ Neighborhood Change Database, which produces US decennial Census and American Community Survey (ACS) data normalized to 2010 tract boundaries (GeoLytics 2021). We estimated annual values with linear interpolation from 1990 to 2000, 2000 to 2010, and 2010 to 2015 (using decennial Census data for all years except 2015, in which we used 2011–2015 5-year ACS estimates). Community characteristics measured at the county level included the average percentage of suicides completed with a firearm during the entire study period as a proxy for firearm ownership prevalence (Azrael et al. 2004) (using mortality data from the California Department of Public Health) and rural-urban status (defined as metro versus non-metro counties using the United States Department of Agriculture’s Rural-Urban Continuum Codes) (USDA Economic Research Service 2020). All community characteristics pertained to the index purchase, and continuous variables were binned into quintiles based on the sample distribution, weighted by population size.

### Dependent variable

Mortality data were obtained from the California Department of Public Health; cases were defined as those who died by firearm suicide (International Classification of Diseases, Tenth Revision codes X72–X74) from January 1, 1996, through December 31, 2015. We used incidence density sampling (Epi R package, version 2.40) to select 5 controls from DROS who were still alive at the time of the case’s death. Under this sampling strategy, we estimate the outcome incidence rate ratio that would be obtained from a cohort study (Vandenbroucke and Pearce 2012; Labrecque et al. 2021). Individuals selected as controls were allowed to subsequently become a case (Rothman et al. 2008). Cases and controls were matched on gender for statistical efficiency. After selecting cases and controls, we determined California residence over time by querying LexisNexis Public Records. Purchasers who were censored because they left the state prior to the case’s death were subsequently excluded from the risk set, and the resulting ratio of cases to controls in the final analytic samples ranged from 1:1 to 1:10.

### Statistical analysis

Data were analyzed with conditional logistic regression (survival R package, version 3.2.3). Cases and controls were compared within risk sets at the time of the case’s death (i.e., controls were censored at the time of the case’s death and subsequent purchases, if any, were not considered in analyses of that risk set). We report unadjusted incidence rate ratios (IRR) and multivariable adjusted incidence rate ratios (aIRR) and 95% confidence intervals (CI) for the independent variables described above (Labrecque et al. 2021). While our goal is descriptive, controlling for transaction and purchaser characteristics is nonetheless useful for more accurately estimating the associations of interest. In the main models, we specified the referent group for categorical variables as the group for which we were most interested in a comparison. We additionally examined all pairwise comparisons for four categorical variables: race/ethnicity, years since prior purchase, handgun category, and handgun caliber.

To capture risk associated with deviation from prior purchasing behavior, we ran additional models in which we interacted independent variables of interest (handgun category, handgun caliber, and inexpensive make) with a 3-level indicator of whether the index purchase for that characteristic differed from any prior purchases: (1) the purchaser had only 1 purchase; (2) the purchaser had more than 1 purchase and the index purchase did not differ from any prior; and (3) the purchaser had more than 1 purchase and the index purchase differed from any prior. In cases of data sparsity, we collapsed this into a 2-level variable: (1) the purchaser had only 1 purchase or the index purchase did not differ from any prior; and (2) the purchaser had more than 1 purchase and the index purchase differed from any prior.

### Sensitivity analysis

We examined the sensitivity of our findings to assigning missing data in race/ethnicity, caliber size, and handgun category to other categories of the variable. Specifically, we re-ran the main model assuming that missing/unknown caliber handguns were small caliber and another model assuming missing/unknown caliber handguns were medium caliber. We did the same with handgun category, assuming all missing/unknown were semiautomatic or revolver, and with race/ethnicity, assuming all missing/unknown were white. In a second sensitivity analysis, we re-ran the main multivariable models adjusting confidence intervals for multiple comparisons using the Ryan–Holm step-down Bonferroni approach (Ludbrook 2000). Finally, given potential concerns regarding collinearity, we re-ran the models in which we interacted handgun characteristics with a 3-level indicator of whether the index purchase differed from any prior purchases, removing the continuous measure of total purchases.

All analyses were conducted in R version 4.0.0 (R Foundation for Statistical Computing, Vienna, Austria). This study was approved by the University of California, Davis Institutional Review Board.

## Results

### Descriptive

During the study period, 390 purchasers died by firearm suicide among those aged 21–25 in 1996, and 512 died by firearm suicide among those aged 50–54 in 1996 (Table 1). Among the younger cohort, the median age of death for cases was 34.5 years (interquartile range [IQR] 28.6, 38.7). The median age of death for cases among the older cohort was 63.7 years (IQR 58.1, 67.8). Median time from the index purchase to the case’s death/censoring was 1.5 years (IQR 0.3, 5.0) among the younger cases, 3.0 years (IQR 1.1, 6.3) among the younger controls, 2.3 years (IQR 0.4, 6.7) among the older cases, and 3.6 years (IQR 1.4, 7.9) among the older controls. A greater percentage of cases than controls in both cohorts were white and a smaller percentage were Hispanic/Latinx. Across cohorts, cases’ index purchase was more often a revolver compared with controls’. Among those aged 50–54 in 1996, distance to dealer for the index handgun purchase was slightly shorter on average for cases than controls.

**Table 1:** Characteristics of cases and controls and their index^a^ handgun purchase, 1996–2015

	Purchasers aged 21–25 in 1996		Purchasers aged 50–54 in 1996	
	Cases	Controls	Cases	Controls
Total, *n* (%)	390 (100)	1594 (100)	512 (100)	2288 (100)
*Characteristics of purchasers*				
Age at first purchase^b^ in years, median (IQR)	27.6 (24.8, 32.2)	25.9 (23.9, 29.5)	56.0 (53.1, 60.8)	54.6 (52.5, 58.6)
Race/ethnicity, *n* (%)				
Asian	45 (11.5)	177 (11.1)	13 (2.5)	95 (4.2)
Black/African American	22 (5.6)	94 (5.9)	14 (2.7)	87 (3.8)
Hispanic/Latinx	65 (16.7)	345 (21.6)	16 (3.1)	120 (5.2)
Other^c^	11 (2.8)	60 (3.8)	16 (3.1)	78 (3.4)
White	247 (63.3)	918 (57.6)	453 (88.5)	1908 (83.4)
Total number of study period purchases, median (IQR)	1.0 (1.0, 2.0)	1.0 (1.0, 2.0)	1.0 (1.0, 2.0)	1.0 (1.0, 2.0)
*Characteristics of index purchase*^*a*^				
Gun show purchase, *n* (%)				
Yes	11 (2.8)	28 (1.8)	9 (1.8)	47 (2.1)
No	379 (97.2)	1566 (98.2)	503 (98.2)	2241 (97.9)
Transaction type, *n* (%)				
Dealer’s record of sale	376 (96.4)	1539 (96.5)	483 (94.3)	2171 (94.9)
Other	14 (3.6)	55 (3.5)	29 (5.7)	117 (5.1)
Handgun category, *n* (%)				
Revolver	91 (23.3)	247 (15.5)	196 (38.3)	750 (32.8)
Semiautomatic	237 (60.8)	1128 (70.8)	251 (49.0)	1226 (53.6)
Other^d^	62 (15.9)	219 (13.7)	65 (12.7)	312 (13.6)
Handgun caliber, *n* (%)				
Small	29 (7.4)	153 (9.6)	88 (17.2)	450 (19.7)
Medium	181 (46.4)	711 (44.6)	243 (47.5)	862 (37.7)
Large^e^	180 (46.2)	730 (45.8)	181 (35.4)	976 (42.7)
Inexpensive handgun^f^, *n* (%)				
Yes	18 (4.6)	61 (3.8)	19 (3.7)	86 (3.8)
No	372 (95.4)	1533 (96.2)	493 (96.3)	2202 (96.2)
Time between index purchase and prior purchase, *n* (%)				
Purchaser had only 1 purchase, or less than 2 years between index purchase and prior purchase	350 (89.7)	1415 (88.8)	464 (90.6)	2040 (89.2)
2–4 years between index purchase and prior purchase	27 (6.9)	108 (6.8)	29 (5.7)	166 (7.3)
5 + years between index purchase and prior purchase	13 (3.3)	71 (4.5)	19 (3.7)	82 (3.6)
Distance to dealer in meters				
Q1	78 (20.0)	336 (21.1)	116 (22.7)	441 (19.3)
Q2	84 (21.5)	321 (20.1)	116 (22.7)	453 (19.8)
Q3	84 (21.5)	308 (19.3)	104 (20.3)	456 (19.9)
Q4	68 (17.4)	316 (19.8)	92 (18.0)	468 (20.5)
Q5	76 (19.5)	313 (19.6)	84 (16.4)	470 (20.5)

*Q* quintile

^a^The purchase closest in time to the case’s death

^b^First purchase observed during the study period

^c^Includes American Indian, Pacific Islander, other (unspecified), and unknown

^d^Includes bolt action, derringer, single-shot, and missing

^e^Includes other and unknown caliber

^f^Proxied by manufacturers producing handguns in the bottom quantile of prices listed in the *Blue Book of Gun Values*

### Associations between purchaser and index purchase characteristics and firearm suicide

In unadjusted models, among both cohorts, older age at first purchase, white race (versus Hispanic/Latinx ethnicity), and the purchase of a revolver (versus semiautomatic pistol) were associated with elevated firearm suicide risk (Table 2). For example, each additional year of age at first purchase was associated with 1.08 (95% CI 1.05, 1.11) times the risk of firearm suicide among the younger cohort and 1.06 (95% CI 1.04, 1.08) times the risk of firearm suicide among the older cohort (Table 2). In addition, among the older cohort, greater distance to retailer was associated with lower risk of firearm suicide, and the purchase of a medium caliber handgun (vs. both small and large caliber) was associated with greater risk (Table 2 footnote).

**Table 2:** Unadjusted associations of purchaser and index^a^ purchase characteristics with firearm suicide among those aged 21–25 and 50–54 in 1996

	Purchasers aged 21–25 in 1996	Purchasers aged 50–54 in 1996
	IRR (95% CI)	IRR (95% CI)
*Characteristics of individuals*		
Age at first purchase^b^ in years	1.08 (1.05, 1.11)	1.06 (1.04, 1.08)
Race/ethnicity		
Asian	0.96 (0.67, 1.37)	0.56 (0.31, 1.01)
Black/African American	0.86 (0.53, 1.40)	0.66 (0.37, 1.17)
Hispanic/Latinx	0.68 (0.50, 0.92)	0.57 (0.33, 0.97)
Other^c^	0.70 (0.36, 1.36)	0.88 (0.51, 1.52)
White	(ref)	(ref)
Total number of study period purchases	0.97 (0.93, 1.02)	0.98 (0.95, 1.01)
*Characteristics of index purchase*^*a*^		
Gun show purchase		
Yes	1.49 (0.73, 3.04)	0.86 (0.42, 1.76)
No	(ref)	(ref)
Type of purchase		
Dealers’ record of sale	(ref)	(ref)
Other	0.99 (0.54, 1.80)	1.09 (0.72, 1.66)
Handgun category		
Revolver	1.78 (1.34, 2.36)	1.30 (1.05, 1.60)
Semiautomatic	(ref)	(ref)
Other^d^	1.32 (0.94, 1.84)	0.97 (0.71, 1.33)
Handgun caliber		
Small	(ref)	(ref)
Medium	1.33 (0.86, 2.05)	1.42 (1.09, 1.86)
Large^e^	1.27 (0.82, 1.96)	0.96 (0.72, 1.27)
Inexpensive handgun^f^		
Yes	1.21 (0.69, 2.11)	1.00 (0.61, 1.65)
No	(ref)	(ref)
Time between index purchase and prior purchase		
Purchaser had only 1 purchase, or less than 2 years between index purchase and prior purchase	(ref)	(ref)
2–4 years between index purchase and prior purchase	0.99 (0.64, 1.55)	0.75 (0.50, 1.13)
5 + years between index purchase and prior purchase	0.63 (0.34, 1.17)	1.00 (0.59, 1.68)
Distance to dealer in meters		
Q1	(ref)	(ref)
Q2	1.14 (0.81, 1.62)	0.99 (0.74, 1.33)
Q3	1.15 (0.81, 1.63)	0.87 (0.65, 1.17)
Q4	0.92 (0.64, 1.32)	0.77 (0.57, 1.05)
Q5	1.02 (0.71, 1.46)	0.69 (0.50, 0.94)

*IRR* incidence rate ratio, *CI* confidence interval, *ref* referent, *Q* quintile

^a^The purchase closest in time to the case’s death

^b^First purchase observed during the study period

^c^Includes American Indian, Pacific Islander, other (unspecified), and unknown

^d^Includes bolt action, derringer, single-shot, and missing

^e^Includes other and unknown caliber

^f^Proxied by manufacturers producing handguns in the bottom quantile of prices listed in the *Blue Book of Gun Values*

When testing all pairwise comparisons, we additionally identified an association between medium versus large caliber handguns among the older cohort (IRR = 1.49; 95% CI 1.20, 1.85)

Among the younger cohort, multivariable model estimates were consistent with those from unadjusted models with the exception of race/ethnicity (Table 3). Among the older cohort, multivariable and unadjusted results were similar; however, in the multivariable model, Black/African American and Asian race (versus white race) were associated with lower risk of firearm suicide, while the purchase of a revolver (versus other handguns) was associated with greater risk of firearm suicide (Table 3 footnote), and there was no longer an association with dealer proximity.

**Table 3:** Adjusted associations of purchaser and index^a^ purchase characteristics with firearm suicide among those aged 21–25 and 50–54 in 1996

	Purchasers aged 21–25 in 1996	Purchasers aged 50–54 in 1996
	aIRR (95% CI)	aIRR (95% CI)
*Characteristics of individuals*		
Age at first purchase^b^ in years	1.07 (1.04, 1.10)	1.05 (1.03, 1.08)
Race/ethnicity		
Asian	0.92 (0.62, 1.37)	0.48 (0.26, 0.89)
Black/African American	0.87 (0.50, 1.50)	0.39 (0.21, 0.73)
Hispanic/Latinx	0.75 (0.53, 1.08)	0.51 (0.29, 0.90)
Other^c^	0.68 (0.34, 1.36)	0.84 (0.45, 1.57)
White	(ref)	(ref)
Total number of study period purchases	0.99 (0.95, 1.03)	0.99 (0.97, 1.01)
*Characteristics of index purchase*^*a*^		
Gun show purchase		
Yes	1.83 (0.85, 3.92)	1.09 (0.51, 2.31)
No	(ref)	(ref)
Type of purchase		
Dealers’ record of sale	(ref)	(ref)
Other	1.21 (0.62, 2.34)	1.04 (0.63, 1.73)
Handgun category		
Revolver	1.78 (1.32, 2.40)	1.36 (1.09, 1.71)
Semiautomatic	(ref)	(ref)
Other^d^	1.21 (0.85, 1.73)	0.88 (0.63, 1.23)
Handgun caliber		
Small	(ref)	(ref)
Medium	1.32 (0.83, 2.10)	1.46 (1.10, 1.94)
Large^e^	1.19 (0.75, 1.90)	0.91 (0.67, 1.22)
Inexpensive handgun^f^		
Yes	1.36 (0.74, 2.51)	1.11 (0.64, 1.91)
No	(ref)	(ref)
Time between index purchase and prior purchase		
Purchaser had only 1 purchase, or less than 2 years between index purchase and prior purchase	(ref)	(ref)
2–4 years between index purchase and prior purchase	1.21 (0.76, 1.95)	0.95 (0.62, 1.47)
5 + years between index purchase and prior purchase	0.80 (0.41, 1.57)	1.39 (0.80, 2.43)
Distance to dealer in meters		
Q1	(ref)	(ref)
Q2	1.14 (0.79, 1.65)	1.01 (0.74, 1.39)
Q3	1.17 (0.80, 1.69)	0.96 (0.70, 1.31)
Q4	0.87 (0.59, 1.27)	0.85 (0.61, 1.17)
Q5	0.99 (0.67, 1.46)	0.78 (0.55, 1.10)

*aIRR* adjusted incidence rate ratio, *CI* confidence interval, *ref* referent, *Q* quintile

^a^The purchase closest in time to the case’s death

^b^First purchase observed during the study period

^c^Includes American Indian, Pacific Islander, other (unspecified), and unknown

^d^Includes bolt action, derringer, single-shot, and missing

^e^Includes other and unknown caliber

^f^Proxied by manufacturers producing handguns in the bottom quantile of prices listed in the *Blue Book of Gun Values*

When testing all pairwise comparisons, we additionally identified an association between medium versus large caliber handguns (aIRR = 1.61; 95% CI 1.27, 2.03) and revolver versus other handguns (aIRR = 1.55; 95% CI 1.10, 2.19) among the older cohort. After adjusting for multiple comparisons within models, the only associations that persisted among the older cohort were for age at first purchase and medium versus large caliber handgun. Results were unchanged for the younger cohort after adjusting for multiple comparisons within models.

We did not find significant interactions for deviation from prior purchasing behavior.

### Sensitivity analysis

Results were not significantly altered as a result of differences in coding missing data or removing the total number of purchases from the models interacting handgun characteristics with the indicator of whether the index purchase differed from any prior purchases. When adjusting for multiple comparisons, results for the younger cohort were consistent with those from the main model (Table 3 footnote). For the older cohort, after adjusting for multiple comparisons, there was no longer an association for race/ethnicity, revolver (versus semiautomatic pistol or other handguns), or medium (versus small) caliber handgun (Table 3 footnote). That is, the only associations that persisted among the older cohort after adjusting for multiple comparisons were for age at first purchase and medium versus large caliber handgun.

## Discussion

This study identified older age at first handgun purchase and the purchase of a revolver (versus semiautomatic pistol) as risk factors for firearm suicide among both cohorts of handgun purchasers. Other variables showed inconsistent associations with firearm suicide risk depending on the cohort and adjustments.

While it is well-established that risk of firearm suicide increases with older age (Wintemute 2015), our study contributes the novel finding that older age at first handgun purchase is associated with elevated firearm suicide risk. Though prior research is limited, our findings for revolvers are consistent with one study showing that the majority (63%) of handguns used in firearm suicides in Sacramento County, CA in the mid-1980’s were revolvers (Wintemute et al. 1988). Nationally and in California, revolvers are somewhat less popular than semiautomatic handguns (Azrael et al. 2017; Kravitz-Wirtz et al. 2019); they are also recovered less often as crime guns (Koper 2014; Wintemute et al. 2004). In our study, revolvers accounted for between 15.5% (younger cohort) and 32.8% (older cohort) of index handgun purchases among the control samples (which provide estimates of the exposure distribution in the source population). Semiautomatic pistols generally hold more rounds of ammunition and are quicker to fire than revolvers and so may have greater perceived or actual utility for most handguns owners, who often own for protection (Azrael et al. 2017; Kravitz-Wirtz et al. 2019).

These findings add to the evidence on firearm suicide risk and may help inform prevention strategies and future research. For example, firearm retailers and range owners in several states have recently partnered with public health researchers and practitioners to prevent suicide through lethal means restriction for suicidal customers and dissemination of suicide prevention materials at their establishments (Polzer et al. 2020). Prior research suggests this may be a promising strategy, as many firearm retailers are willing to learn about how they can prevent firearm suicide and engage in firearm suicide prevention interventions with their customers (Walton and Stuber 2020; Vriniotis et al. 2015). Our results indicate that, in addition to standard epidemiologic risk factors for suicide, firearm retailers engaged in these efforts may consider the purchase of a revolver as an indicator of firearm suicide risk. Findings for older age at first purchase may be less immediately actionable when comparing purchasers of different birth cohorts since we identified the association even among the younger cohort (the median age of first purchase among cases in the younger cohort was 27.6 years).

Previous research suggests some people purchase a firearm for the purpose of suicide (Studdert et al. 2020; Wintemute et al. 1999). While we cannot measure suicidal intent, it is possible that those who purchased their first handgun at older ages relative to their peers or who did not purchase a handgun often associated with common firearm uses, such as self-protection or sport (e.g., a semiautomatic pistol), are at greater risk of firearm suicide. A more direct understanding of purchasing preferences when purchasing with the intent of firearm suicide would be informative, as the implications for suicide prevention may be different for purchasers intending to die by suicide versus those with other motivations or intentions for use.

Some differences in findings between the older and younger cohorts are suggestive of cohort or age effects in purchasing patterns. In addition to age at first purchase and the purchase of a revolver, risk factors among the older cohort included the purchase of a medium caliber handgun, white race, and, in the model without covariate adjustment, distance to dealer. These findings are consistent with research showing that older white men are at greatest risk of firearm suicide (Wintemute 2015). The association for distance to dealer suggests that older individuals who died by firearm suicide either lived closer to firearm dealers at the time of purchase on average or were less inclined to travel to obtain a firearm than their peers who did not die by firearm suicide. However, findings for race/ethnicity, handgun type, and caliber among the older cohort were sensitive to adjusting for multiple comparisons. Among the younger cohort, there was no association with handgun caliber, and race/ethnicity (white race versus Hispanic/Latinx) was only associated with firearm suicide risk in models without covariate adjustment. Differences between cohorts could also be due to “missing” data on earlier life purchasing and survivor bias among the older cohort.

That we are not able to identify many handgun purchaser and transaction characteristics as markers of firearm suicide risk may be because handgun ownership in general increases firearm suicide risk. That is, because firearm owners as a group are at higher risk of firearm suicide than are non-firearm owners, purchaser and transaction characteristics may provide minimal information on risk *among* firearm owners. Our null findings may also be because of limited power to detect differences. Future research that considers these purchaser and transaction characteristics in various combinations with each other, with more data, and in more flexible models—such as machine-learning risk prediction—may be useful for identifying specific handgun purchasers at greatest risk.

### Limitations

Our study should be considered in light of several limitations. Though California implemented a comprehensive background check policy in 1991, which requires almost all sales to be conducted through a licensed firearm retailer, firearm transaction records may nonetheless be incomplete and subject to measurement error. In addition, data are not representative of long gun sales (which were only recorded beginning in 2014) or illegal acquisitions. California has comparatively stringent restrictions on firearm purchase and possession, and results from the current study may not generalize to other states.

We cannot determine whether the specific handguns purchased by cohort members were those used in suicide. Furthermore, our study is descriptive, and we did not control for correlates of suicide such as individual’s mental health, which may have confounded our results. However, our intent was to identify risk factors and we make no assumptions of causality. Future research should examine the broader individual and social contexts that contributed to the associations we observed.

Lastly, our study period only extends through 2015, though we believe results would be similar had we included more recent data at least up to the coronavirus pandemic beginning in 2020. There was a substantial surge in firearm purchasing during the pandemic and preliminary data suggest that pandemic-era purchasers may have been different (e.g., in race/ethnicity, gender, age, and motivation) than non-pandemic-era purchasers (Tavernise 2021).

## Conclusion

Firearm access is a well-established risk factor for firearm suicide. In this nested case–control study of legal handgun purchasers in California, we identified specific characteristics of handgun transactions and purchasers associated with firearm suicide risk, including older age at first purchase and the purchase of a revolver.

## Data Availability

The data that support the findings of this study are available from the California Department of Justice and California Department of Public Health but restrictions apply to the availability of these data, which were used under license for the current study, and so are not publicly available.

## Abbreviations

CA DOJ: California Department of Justice
DROS: Dealer’s Record of Sale
ACS: American Community Survey
IRR: Incidence rate ratio
aIRR: Adjusted incidence rate ratios
CI: 95% confidence intervals
